# Environmental drivers of date fruit unripening syndrome in dryland regions of Saudi Arabia

**DOI:** 10.3389/fpls.2025.1587061

**Published:** 2025-09-02

**Authors:** Zafar Iqbal, Muhammad Munir, Adil Alshoaibi, Nashi Khalid Alqahtani

**Affiliations:** ^1^ Central Laboratories, King Faisal University, Al-Ahsa, Saudi Arabia; ^2^ Date Palm Research Center of Excellence, King Faisal University, Al-Ahsa, Saudi Arabia

**Keywords:** date palm, date fruit unripening syndrome (DFUS), temperature, Khalas, UV index, humidity, Saudi Arabia

## Abstract

Date Fruit Unripening Syndrome (DFUS), a very recently observed phenomenon, is a devastating non-ripening disorder hindering the Rutab-to-Tamar transition. While observed sporadically since 2019, DFUS became prominent in 2024, disproportionately affecting the economically important Khalas cultivar (40%), followed by Sheshi (19%), Reziz and Ghur (16%), and Shahal (10%) in Al-Ahsa. With the highest incidence in Al-Ahsa (20-30%), followed by Riyadh (10-15%) and Al-Qassim (5-10%), DFUS significantly reduces fruit quality and yield, threatening farmers’ livelihoods and the socio-economic stability of date palm-dependent communities. This pioneering study investigated the impact of environmental factors—temperature, relative humidity, solar UV index, heat units, and irradiance—on date palm fruit ripening in Al-Ahsa, Saudi Arabia, from 2019 to 2024, employing ANOVA (*p* ≤ 0.05) and Duncan’s Multiple Range Test for robust statistical analysis. Significant inter-annual temperature variations, including daytime extremes up to 48 °C during Kimri and Khalal stages, potentially disrupted fruit metabolism and ripening. Relative humidity (RH) ranged widely (5-96%), with the Khalal stage particularly sensitive to low humidity, while high RH during the Rutab stage (2023/2024) may have hindered the necessary moisture loss thus negatively impacting the fruit ripening. Increased solar UV exposure, particularly during the Khalal and Rutab stages, may have contributed to DFUS by disrupting fruit pigment synthesis and inducing oxidative stress. Total heat unit accumulation peaked in 2024, while total irradiance declined, potentially hindering sugar conversion and softening during Rutab. Through meticulous field observations and environmental data analysis, we identified critical stressors—such as temperature fluctuations, elevated heat units, and high solar UV indices—as key drivers of DFUS. These findings provide crucial insights into the mechanisms behind DFUS, enabling the development of targeted interventions, such as optimized agricultural practices and potentially genetic solutions, to mitigate the syndrome and protect palm productivity.

## 1 Introduction

Date palm (*Phoenix dactylifera* L.), a perennial woody plant remarkably adapted to arid environments, is cultivated for millennia and has been integral to the economies, food security, and cultural heritage of Middle Eastern and North African countries (Krueger, 2021). Renowned for its ability to thrive in saline soils, the date palm requires substantial water for optimal growth, making it a vital ecological asset in regions prone to desertification. Beyond its nutritional value, date palm fruits are rich in bioactive compounds with antioxidant and anti-inflammatory properties, offering significant health benefits (Zhang et al., 2013). Globally, date production has reached an impressive ~9.75 million tons annually, with Egypt, Saudi Arabia, and Iran leading the charge, underscoring its dual role as a staple food and a critical export commodity (Faostat, 2024). Sustainable date palm cultivation is therefore essential for the long-term economic viability and livelihood security of communities reliant on this valuable resource.

Date palm cultivation holds immense economic, cultural, and agricultural significance in Saudi Arabia, which ranks among the world’s top date producers. The Kingdom cultivates approximately 400 varieties across 156,000 hectares, yielding around 1.61 million tons annually (El-Habba and Al-Mulhim, 2013; FAOStat, 2024). Popular cultivars, such as Khalas, Reziz, and Sheshi, are highly prized by consumers, especially in the Eastern Province (Al-Fuhaid et al., 2006). Recognizing the crucial role of date palm, Saudi Vision 2030 prioritizes its production for economic diversification, food security, and the promotion of tourism and cultural heritage.

In Saudi Arabia, the Al-Ahsa oasis is a prominent agricultural hub, renowned for its extensive date palm cultivation. Despite the surrounding arid landscape, Al-Ahsa’s oases thrive, supporting around 3–4 million date palm trees across 8,200 hectares (Almutawa, 2022). The region’s dry tropical climate features scorching summers (35-45 °C, occasionally exceeding 50 °C) and relatively cold winters (2-22 °C) (Fathi and Al-Kahtani, 2009; Allbed et al., 2014). The soil is predominantly sandy loam, with significant areas affected by salinity (Biro et al., 2020). Within this oasis, date palm trees occupy roughly 70% of the cultivated area (Almadini et al., 2021), featuring 40 distinct cultivars, with Khalas, Reziz, Sheshi, Ghur, and Shahal being the most prevalent and highly valued for their unique processing characteristics, appealing flavors, and vibrant colors (Elsabea, 2012; Elsebaei, 2021). Saudi Arabia’s arid climate makes it highly vulnerable to climate change, threatening date palm cultivation. The country emitted 623 million metric tons of CO_2_ in 2023, exacerbating global warming. Al-Ahsa, in the Eastern Province, is a climate hotspot, with models predicting temperature rises of 3–6 °C by 2100 (Al Zawad and Aksakal, 2010; Sharif, 2015). Relative humidity is projected to decline by 0.8–2.5% by 2050 (Almazroui, 2013; Chowdhury and Al-Zahrani, 2013). These changes may reduce climatic suitability for date palms, altering cultivar viability.

**Figure 1:**
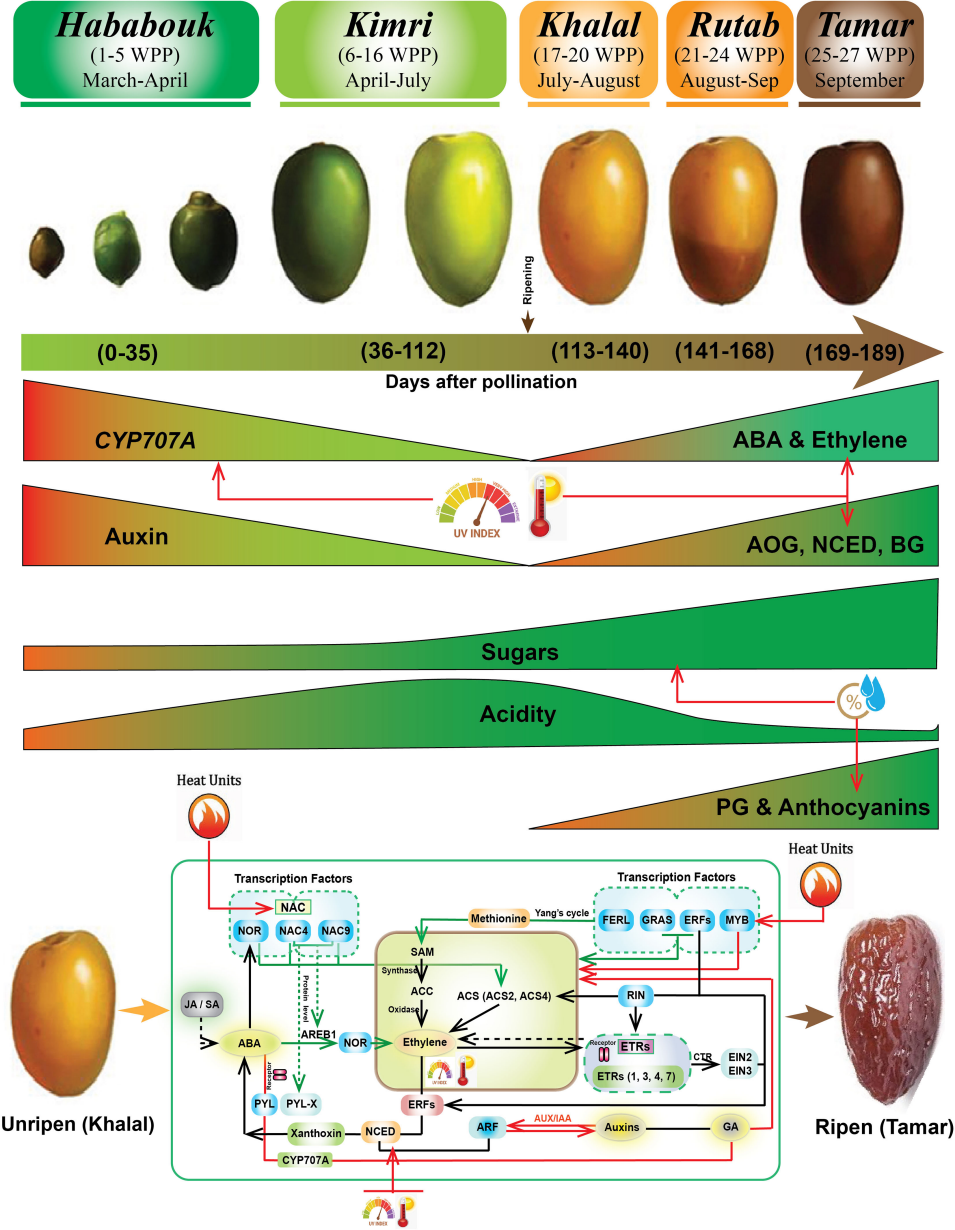
Biochemical and molecular events during date palm fruit ripening. The biochemical and molecular changes during date palm fruit ripening stages (Hababouk, Kimri, Khalal, Rutab, Tamar), shown with representative fruit images and a timeline in weeks post-pollination (WPP), approximate months, and cumulative days. Key biochemical changes (enzyme roles: CYP707A, AOG, NCED, BG, PG; hormones: Auxin, ABA, Ethylene; sugars, acidity, anthocyanins) and environmental influences (Heat Units, UV Index) are highlighted. The lower panel reiterates the core molecular regulatory network (transcription factors: NAC, NOR, ERFs, GRAS, MYB, RIN; hormones: ABA, ethylene, auxins, GA, JA/SA; signaling pathways) from **Figure 8**.

Date palm fruits undergo five key maturation stages: Hababouk, Kimri, Khalal, Rutab, and Tamar (**Figure 1**) (Al-Mssallem et al., 2013). During these five stages, date fruit color changed due to the interplay of chlorophyll and carotenoids (chloroplasts/chromoplasts) and phenolic pigments (anthocyanins, flavanols, proanthocyanins; vacuoles) (Lancaster et al., 1997). Green and yellow hues (epicarp) are due to chlorophyll and carotenoids, respectively, while brown is primarily from anthocyanins. During ripening, chlorophyll/carotenoids degrade, and anthocyanins are synthesized (Hu et al., 2000), resulting in a color shift from green to yellow (Kimri and Khalal) to brown (Rutab and Tamar). The transition from Khalal to Rutab is particularly critical, marked by higher anthocyanins contents and significant biochemical changes, including increased sugar content, fluctuations in antioxidant capacity and phenolic content (Al-Mssallem et al., 2013; Haider et al., 2013; Barakat and Alfheeaid, 2023). These changes are influenced by cultivar-specific traits and environmental conditions, underscoring the importance of optimizing horticultural practices to enhance fruit quality and nutritional value (Al-Alawi et al., 2017).

**Figure 2:**
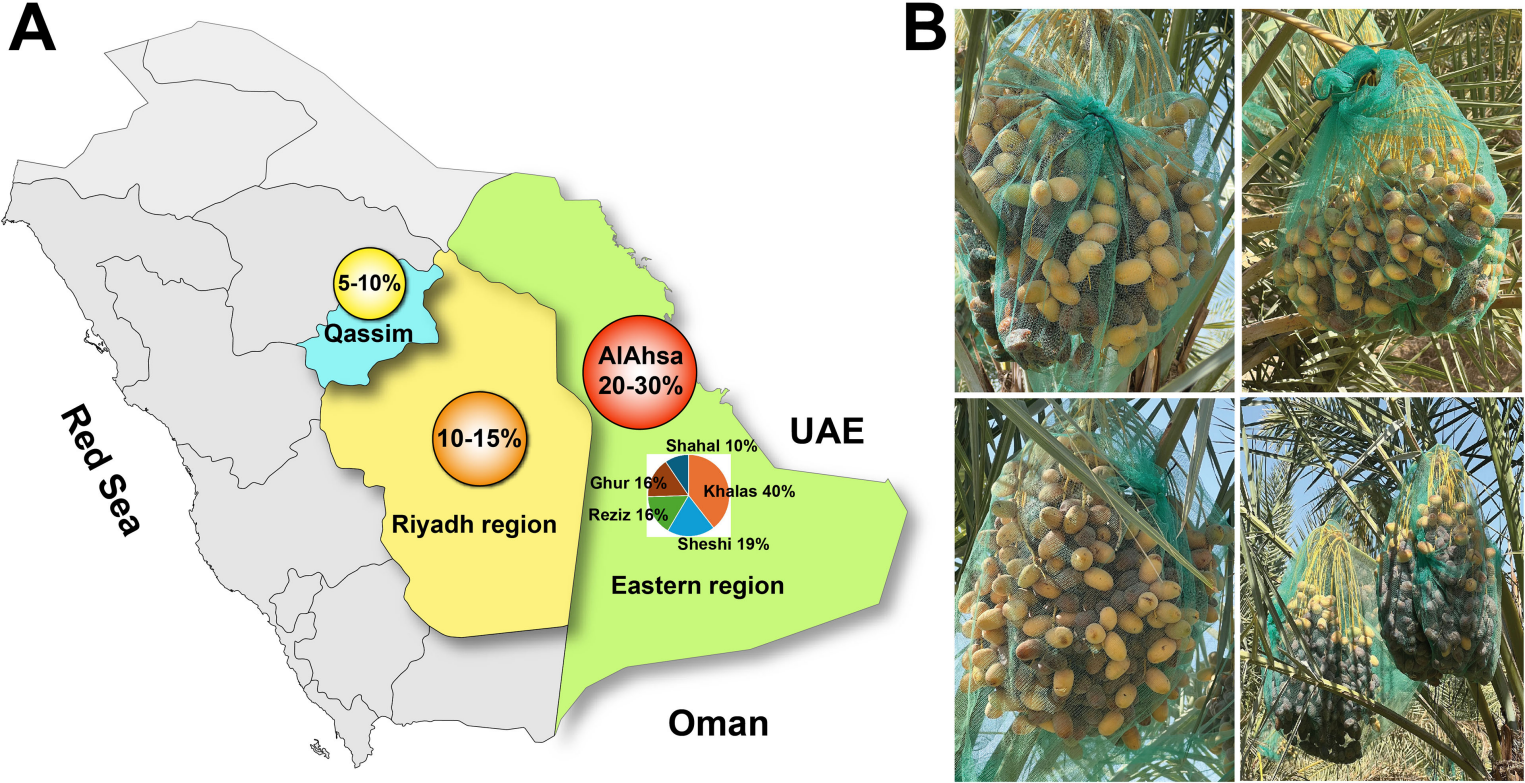
Distribution of date fruit unripening syndrome (DFUS) across major date‐growing regions of Saudi Arabia **(A)** and field images of affected date bunches **(B)**.

Date palm cultivation in Saudi Arabia, despite its ecological and economic importance, faces numerous challenges, including water scarcity, pest infestations, and the impacts of climate change (Allbed et al., 2017; Alotaibi et al., 2021; Almutawa, 2022). Among these challenges, the emergence of Date Fruit Unripening Syndrome (DFUS)—a recently identified disorder—poses a significant and growing threat to date production. First observed in Al-Ahsa in 2016, DFUS is characterized by the failure of date fruits to ripen beyond the Khalal (transition from Rutab to Tamr) stage. Affected fruits retain their yellow coloration, exhibit reduced sugar content, and develop an altered texture, severely impacting their marketability and yield. DFUS affects prominent date palm cultivars such as Khalas (40%), Reziz (19%), Sheshi (16%), Ghur (16%), Shahal (10%), and some other cultivars, with its prevalence varying regionally. During 2024, the Eastern Province (Al-Ahsa) reported the highest incidence (20-30%), followed by Riyadh (10-15%) and Al-Qassim (5-10%) (personal communication with farmers) (**Figure 2A**). Unfortunately, no economic impact of DFUS has been capitalized till the conductance of this study. DFUS is likely driven by environmental stressors and poses a significant threat to date production, leading to substantial economic losses. Similar to DFUS, Stay-Green Syndrome (also known as Zhengqing in China) and Green Stem Syndrome is affecting various crops (such as soybean and sorghum) in the world (Sakuraba et al., 2015; Li et al., 2019). SGS is characterized by delayed senescence, where persistent chlorophyll retention in leaves and stems leads to impaired nutrient remobilization and yield losses due to incomplete pod or grain maturation. This condition is typically induced by abiotic stressors (e.g., drought, nutrient imbalances) or hormonal dysregulation, particularly cytokinin overaccumulation (Zhang et al., 2016; Wang et al., 2025). In contrast, DFUS specifically targets fruit tissues, disrupting key ripening processes—such as sugar accumulation and cell wall softening—while leaving vegetative growth unaffected. This suggests a localized physiological disorder, potentially involving ethylene signaling or date palm-specific cell wall-modifying enzymes. Further distinctions emerge when comparing DFUS to other ripening disorders: unlike genetically stable mutants such as tomato nor, rin, and cnr (Wang et al., 2020), DFUS occurs sporadically, complicating its predictability and management. For date fruit ripening, optimal ripening conditions, although vary among cultivars, require a certain amount of heat units (1100–3990 hours) above 18 °C with minimal precipitation (Zaid and De Wet, 2002; Lobo et al., 2013). High humidity, especially during flowering or later stages of fruit development, can lead to physiological disorders and limit commercial production (Yahia, 2011).

Date fruit ripening, governed by ethylene and ABA signaling, is highly vulnerable to climate stressors that may trigger DFUS. The date palm’s complex genome (~750 megabase pairs and over 30,000 genes) (Hazzouri et al., 2019; Alhajhoj et al., 2022; Iqbal and Munir, 2024) encodes key ripening regulators: ethylene activates polygalacturonase (PG) for tissue softening (Giovannoni, 2001; Wang et al., 2018), while ABA drives sugar accumulation via sucrose transporters (Gupta et al., 2022; Wu et al., 2023). Carbohydrates, particularly sugars like glucose and fructose, accumulate during ripening, increasing fruit sweetness and influencing subsequent metabolic changes. Polyphenol oxidase (PPO) further contributes to fruit quality by mediating browning reactions, which can affect appearance and marketability (Eid et al., 2011, 2013; Mishra and Gautam, 2016). Heat, UV radiation, and humidity fluctuations—worsened by climate change—can disrupt ethylene and ABA biosynthesis or signaling, impairing enzyme activation and sugar transport. Such disruption likely underlies DFUS. This link highlights the need to investigate how abiotic stress alters ripening at the molecular level and supports an environmental focus in DFUS research.

This study aimed to unravel the underlying causes of DFUS by investigating the role of environmental variables. Focusing on Al-Ahsa region of Saudi Arabia, this research explores how environmental factors, including temperature, RH, solar UV index, heat units, and irradiance, impact the physiological and molecular mechanisms governing fruit ripening. To the best of our knowledge, this is the first comprehensive study to address the DFUS issue in date palms. The findings from this study provide critical insights that could serve as a foundation for developing targeted interventions, including optimized agricultural practices and potential genetic solutions, to mitigate DFUS and ensure sustainable date palm productivity in the face of changing climatic conditions. This pioneering work not only fills a significant knowledge gap but also offers practical strategies to address a pressing agricultural challenge, with implications for both local and global date palm cultivation.

## 2 Materials and methods

### 2.1 Date palm non-ripening data collection

To assess the prevalence of DFUS, a multi-pronged approach was employed during June-September 2024, combining direct field surveys in Al-Ahsa and farmer interviews elsewhere in Saudi Arabia. In Al-Ahsa, farms were randomly selected with ~4–5 km spacing, and direct observations were made and photographed regarding fruit development, proportion of affected trees, and palm health. Outside Al-Ahsa, logistical constraints necessitated contacting local farmers by phone to gather information on non-ripening incidence, affected cultivars, estimated extent, and perceived causes. This qualitative data, derived from farmer self-reports, offered valuable regional context and enriched the Al-Ahsa field data. Despite potential recall bias and subjectivity—especially in estimating yield losses and DFUS incidence—efforts to minimize inaccuracies included a validation filter, excluding outlier claims (e.g., yield losses >50%) unless corroborated by at least two independent farms in the same region.

### 2.2 Environmental data collection

To evaluate the influence of environmental factors on date palm cultivation, we obtained comprehensive climatic data for the period spanning 2019 to 2024 of Al-Ahsa region. This dataset included temperature metrics (minimum, maximum, and average), ultraviolet (UV) index, heat units, average solar radiation, and humidity levels (minimum, maximum, and average). The data were sourced from NASA’s POWER (Prediction of Worldwide Energy Resource) publicly accessible climate database (POWER Data V10) (https://power.larc.nasa.gov/data-access-viewer/). Temperature, relative humidity, solar irradiance, and UV index parameters are provided at a consistent spatial resolution of 0.5° × 0.5° (≈55 km at the equator). The climate variables were accessed at ‘single point’ location (25°.0”N, 49°.3”E) and at ‘hourly’ temporal level from 2019-2024. Temperature and relative humidity were recorded at two meters from the surface whereas solar irradiance, and UV index were recorded at ground level. The meteorological parameters are based upon the MERRA-2 (Modern-Era Retrospective Analysis for Research and Applications, Version 2) assimilation model using the GEOS-5 atmospheric model, validated against ground stations with mean uncertainties of ±0.5 °C (temperature), ± 5% (relative humidity), ± 5–10% (solar irradiance), and ±0.5 (UV Index). In order to ensure the reliability and consistency of the NASA’s data, the climate data was also obtained from the weather station installed at Al-Ahsa International Airport (ICAO: OEAH, 25°17’37.0”N 49°29’10.9”E), which provides real-time weather information of the region. Both data sources showed strong consistency between the two sources datasets, supporting the use of NASA POWER data as a reliable input. These environmental parameters were analyzed to identify their potential impact on date palm fruit growth and development, and the occurrence of DFUS during the experimental period.

### 2.3 Data analysis

Collected field data (palm health, fruit development) and retrieved environmental data (temperature, humidity, solar UV, heat units, and irradiance) were organized and categorized for the subsequent analyses. Climate data (2019–2024) were partitioned into four distinct pre-ripening stages of date palm fruit development: Hababouk (21 March–24 April; 35 days), Kimri (25 April–10 July; 77 days), Khalal (11 July–7 August; 28 days), and Rutab (8 August–4 September; 28 days). Sample sizes corresponded to stage durations (Hababouk: n = 35; Kimri: n = 77; Khalal: n = 28; Rutab: n = 28). Given interannual variability in diurnal and nocturnal climate conditions, we statistically compared the effects of climatic variables (temperature, relative humidity, UV index) across fruit development stages using ANOVA and DMRT. The Rutab stage—identified as the most vulnerable phase during the unripe-to-ripe transition—is particularly sensitive to adverse climatic conditions. Consequently, we calculated year-wise (2019–2024) total heat units and irradiance specifically for the Rutab stage. These cumulative metrics were then analyzed statistically via ANOVA and DMRT tests.

Statistical analyses were performed using Genstat software (14th Edition, VSNi, Hemel Hempstead, England). To evaluate significant differences, Analysis of Variance (ANOVA) was conducted, with a significance threshold set at *p* ≤ 0.05. For cases where ANOVA indicated significant differences, Duncan’s Multiple Range Test (DMRT) was employed to compare and determine the significance between the means. OriginPro v2024 was used for all graphical representations, visualizing environmental trends and correlations with non-ripening incidence (https://www.originlab.com/).

Effect sizes (η^2^) were calculated to quantify the proportion of variance in date palm fruit developmental stages explained by individual environmental variables. One-way Analysis of Variance (ANOVA) was performed for each environmental factor across the four fruit stages (Hababouk, Kimri, Khalal, and Rutab). The η^2^ values were derived using the ratio of the sum of squares for the effect (SS_effect) to the total sum of squares (SS_total), following the formula η^2^ = SS_effect/SS_total. These calculations were conducted using Genstat software, and interpreted based on conventional benchmarks for small (0.01), medium (0.06), and large (0.14) effects as proposed by Cohen (2013) (Cohen, 2013).

## 3 Results

### 3.1 Effect of temperature

**Figure 3:**
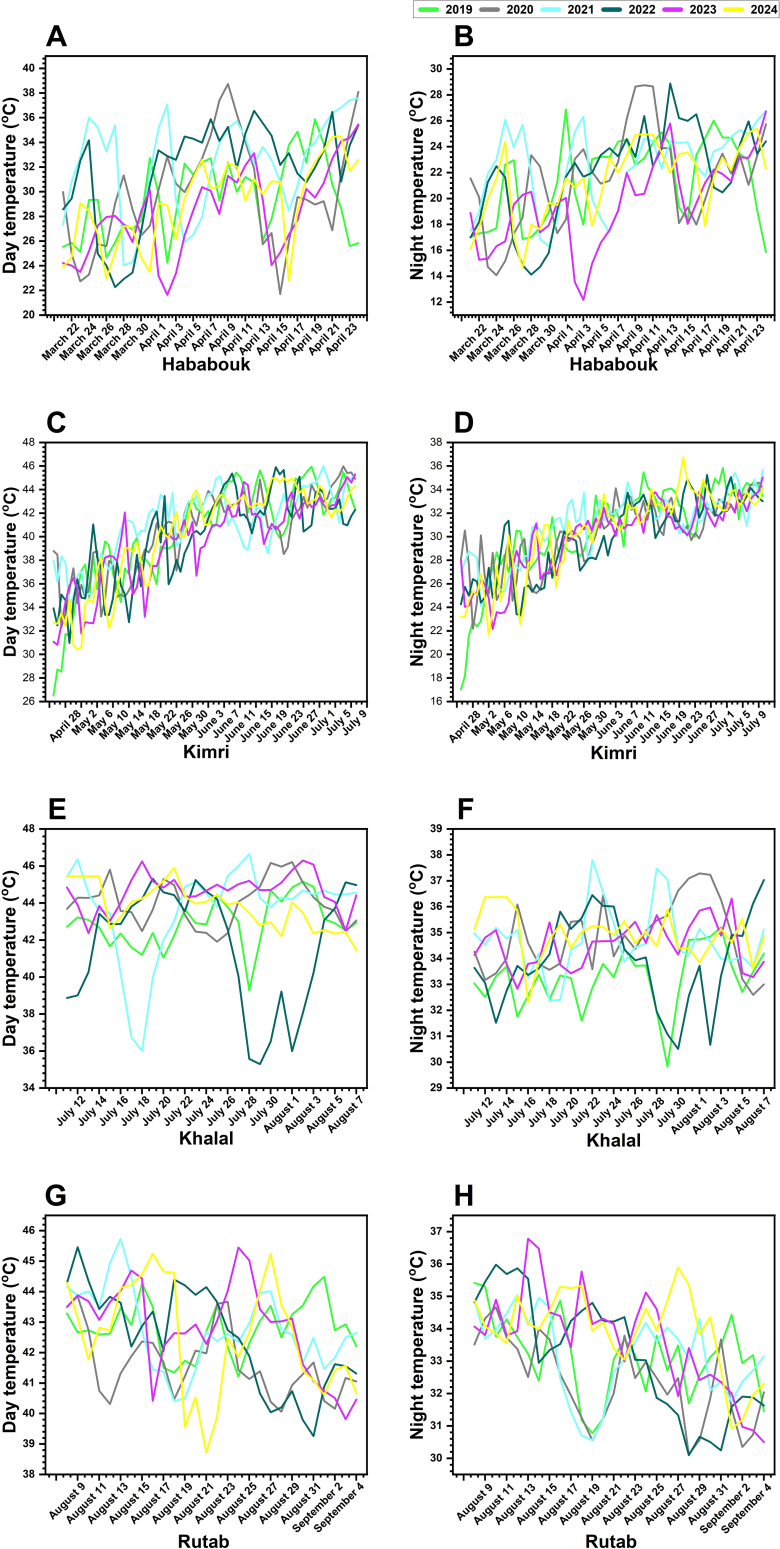
Inter-annual variations in day and night temperatures across different ripening stages of date palm fruit from 2019 to 2024 in Al-Ahsa, Saudi Arabia. **(A, B)** Temperature fluctuations during the Hababouk stage. **(C, D)** Temperature trends during the Kimri stage. **(E, F)** Temperature changes during the Khalal stage. **(G, H)** Temperature variations during the Rutab stage. Each colored line represents a specific year: 2019 (green), 2020 (gray), 2021 (cyan), 2022 (blue), 2023 (magenta), and 2024 (yellow). The x-axis denotes the date (month and year), while the y-axis indicates temperature (°C).

The day and night temperature graph (**Figure 3**) illustrates the fluctuations in temperatures during the ripening stages of date palm fruit over a six-year period, from 2019 to 2024, in Al-Ahsa, Eastern Province, Saudi Arabia.

In the Hababouk, an early stage of fruit development, day temperatures range from approximately 24 °C to 38 °C, with noticeable variations among the years (**Figure 3A**). The years 2020 and 2021 exhibit higher fluctuations, whereas 2023 and 2024 show more stable trends. Night temperatures in this stage vary from 14 °C to 28 °C, with 2020 and 2021 displaying significant peaks, suggesting potential heat stress during early fruit development (**Figure 3B**). Notably, the temperatures in 2024 seem to be slightly lower compared to the previous years.

During the Kimri stage, day temperatures show a steady increase, reaching up to 48 °C in some years (**Figure 3C**). Notably, 2020 and 2021 experience the highest temperature spikes, whereas 2024 follows a more stable pattern. Night temperatures rise gradually from 18 °C to 34 °C, with 2020 and 2021 showing more extreme fluctuations (**Figure 3D**). These temperature peaks during the Kimri stage could impact fruit metabolism and development, possibly contributing to irregular ripening patterns. There was a noticeable peak in day temperatures around June in most years. Again, 2024 appears to have slightly cooler temperatures compared to some of the earlier years.

The Khalal stage is characterized by the fruit reaching full size but still being unripe. Day temperatures (**Figure 3E**) show a high degree of variability, with peaks reaching above 40 °C in some years. shows day temperatures fluctuating between 36 °C and 48 °C, with 2021 and 2022 exhibiting considerable variability. In contrast, 2024 displays a smoother trend, suggesting more stable climatic conditions. Night temperatures (**Figure 3F**) remain within the range of 29 °C to 38 °C, with 2021 and 2020 showing irregular spikes, possibly leading to uneven ripening. The relatively stable night temperatures in 2023 and 2024 may promote better fruit maturation.

Finally, the Rutab stage exhibits day temperatures fluctuating between 38 °C and 46 °C, with 2020 and 2021 showing higher variability (**Figure 3G**). Like previous stages, 2024 maintains a more moderate trend, indicating a potentially more favorable climate for fruit ripening. Night temperatures in this stage range from 30 °C to 37 °C, with 2020 and 2021 displaying high peaks, which could impact fruit softening and sugar accumulation (**Figure 3H**). Again, 2023 and 2024 appear to have more stable nighttime temperatures, which may facilitate improved fruit quality.

Overall, the **Figure 3** suggests that there are significant inter-annual variations in both day and night temperatures during the date palm fruit ripening process. These temperature fluctuations, particularly during the critical Khalal stage, could potentially influence the ripening process and the overall quality of the dates. The apparent lower temperatures in 2024 in some of the stages might indicate a shift in climatic patterns that could affect future yields. It is important to note that this is a visual analysis, and further statistical analysis would be needed to confirm these observations and explore the relationship between temperature and fruit ripening in detail. Also, the **Figure 3** does not explicitly mention the exact temperature values, making it difficult to provide a more precise description of the temperature ranges.

### 3.2 Effect of relative humidity

**Figure 4:**
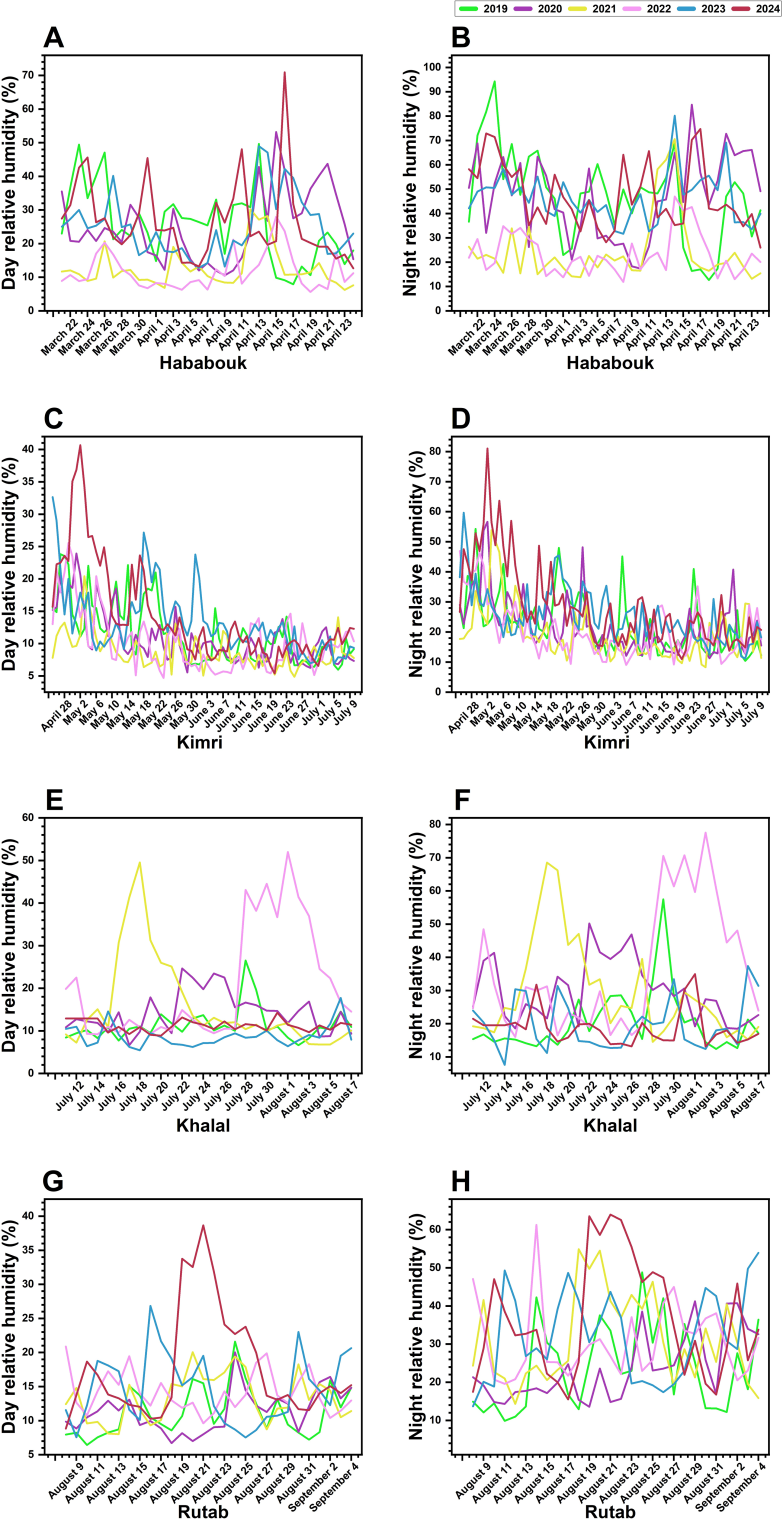
Inter-annual variations in day and night relative humidity (RH; %) across different ripening stages of date palm fruit from 2019 to 2024 in Al-Ahsa, Saudi Arabia. **(A, B)** RH fluctuations during the Hababouk stage. **(C, D)** RH trends during the Kimri stage. **(E, F)** RH changes during the Khalal stage. **(G, H)** RH variations during the Rutab stage. Each colored line represents a specific year: 2019 (green), 2020 (gray), 2021 (cyan), 2022 (blue), 2023 (magenta), and 2024 (red). The x-axis denotes the date (month and year), while the y-axis represents RH (%).

Relative humidity (RH) is a critical factor influencing the ripening stages of date palm fruits. Analysis of RH during date palm fruit ripening in Al-Ahsa revealed notable inter-annual variations across all developmental stages (Hababouk, Kimri, Khalal, and Rutab) from 2019 to 2024. The RH values range from 5% to 96%, indicating a wide spectrum of humidity conditions that the fruits were exposed to during the study period (**Figure 4**).

During the Hababouk stage, day RH exhibited considerable variability with distinct peaks and troughs, indicating fluctuating atmospheric moisture. Night RH also fluctuated but appeared less erratic compared to daytime conditions. Both day and night RH showed a general upward trend from March to May, consistent with seasonal warming (**Figures 4A, B**).

In the Kimri stage, a similar pattern of RH fluctuations was observed. Day RH continued to show significant inter-annual variations, while night RH varied less dramatically (**Figures 4C, D**). Notably, during 2023, mid-May to end-May the RH remained high. Likewise, during 2024, RH level remained high at the end of April to mid of May, potentially impacting the fruit’s growth rate and metabolic activities. RH levels during this stage appeared slightly lower compared to Hababouk.

The Khalal stage was characterized by a wide range of day and night RH fluctuations, including extremely low levels in some years. Notably, for the year 2023 and 2024, RH levels showed less fluctuations compared to the other years (**Figures 4E, F**). The data suggests that the Khalal stage may be particularly vulnerable to variations in RH, as low humidity levels could affect the fruit’s water retention and physiological processes.

Finally, at the last Rutab stage (**Figures 4G, H**), unlike the Khalal stage, day RH showed considerable variability with distinct peaks and troughs during the year 2023 and 2024, indicating fluctuating atmospheric moisture, though the fluctuations appear less pronounced compared to the Khalal stage. Although similar trends were observed at night RH but other years like 2021 and 2022 showed fluctuation. The data suggests that RH may play a less critical role during the Rutab stage compared to earlier developmental phases. However, the observed fluctuations could still influence the final ripening process, particularly in terms of fruit texture and sugar accumulation.

The results highlight significant inter-annual variations in both day and night RH across all stages of date palm fruit development. The data reveals that the later stages (Khalal and Rutab) may particularly be sensitive to fluctuations in RH, with potential impacts on fruit growth and development. These two fruit development stages, characterized by extreme variability in day RH, appear to be the most vulnerable to environmental moisture changes, which could affect fruit texture softening and quality.

### 3.3 Effect of solar UV index

**Figure 5:**
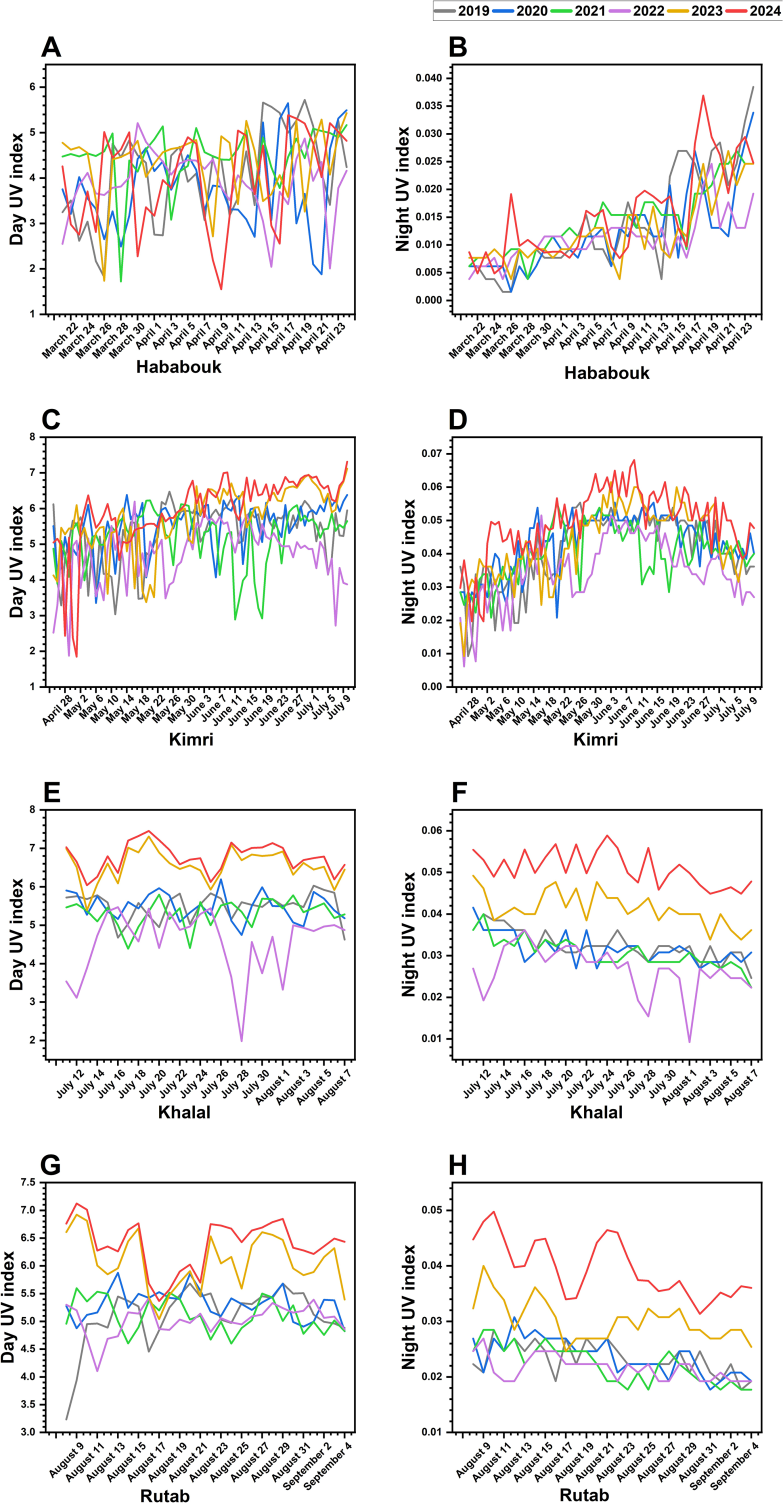
Inter-annual variations in solar UV index during date palm fruit ripening (2019-2024) in Al-Ahsa, Saudi Arabia. **(A, B)** UV index fluctuations during the Hababouk stage. **(C, D)** UV index trends during the Kimri stage. **(E, F)** UV index changes during the Khalal stage. **(G, H)** UV index variations during the Rutab stage. Each colored line represents a specific year: 2019 (gray), 2020 (blue), 2021 (green), 2022 (purple), 2023 (yellow), and 2024 (red). The x-axis represents the date (month and year), while the y-axis indicates the UV index.

The effect of solar UV radiation on date fruit ripening varies significantly (*p* ≤ 0.05) across years and developmental stages, indicating the potential influence of changing environmental conditions. Over the years, a noticeable trend of increasing UV exposure, both during the day and at night, has been observed (**Figure 5**).

In the early Hababouk stage, the daytime UV index showed a general increase from March to May, with noticeable inter-annual variations (**Figure 5A**). For instance, 2020 and 2022 show relatively higher UV indices compared to other years. Night UV indices (**Figure 5B**), as expected, are significantly (*p* ≤ 0.05) lower and show less dramatic fluctuations, though a slight upward trend is visible alongside the day UV increase.

In the Kimri stage, day UV indices (**Figure 5C**) continue to show fluctuations and variations between years. However, the magnitude of variation seems slightly less compared to the Hababouk stage. Night UV indices (**Figure 5D**) remain low with minor fluctuations. Notably, both day and night UV indices exhibited a steady increase, particularly in recent years (2023 and 2024). Higher UV exposure during this stage may influence biochemical pathways associated with fruit growth and metabolic changes leading to ripening.

The Khalal stage, known for its color change and sugar accumulation, experienced peak daytime UV levels in 2023 and 2024 (**Figures 5E, F**). During these years, UV indices reach higher levels compared to the previous stages and show more pronounced peaks, indicating a period of greater exposure to UV radiation. Notably, three consecutive years, 2019-2021, UV index exhibited similar trends, with just a few small fluctuations. Nonetheless, during 2022, a highly abrupt decrease in UV was noted. Night UV indices (**Figure 5F**) showed some increase compared to earlier stages but remain considerably lower than day values. Such high UV index, during the recent years (2023-2024) night potentially affecting respiration and sugar metabolism, leading to DFUS problem.

The Rutab stage exhibited the highest UV exposure in recent years (**Figures 5G, H**). Daytime UV values were significantly (*p* ≤ 0.05) elevated in 2023 and 2024. Interestingly, while the night UV index followed an increasing trend throughout the earlier stages, it showed a slight decline during Rutab, indicating a stabilization phase as ripening reaches completion. The combined effect of increased UV exposure, particularly in the later developmental stages, suggests that ripening dynamics may be influenced by external environmental factors such as rising temperatures and prolonged sunlight exposure.

Overall, the increasing trend in UV exposure over the years, especially during the Kimri, Khalal, and Rutab stages, suggests that climatic changes could play a role in altering the natural ripening process of date palm fruit. Higher UV levels in the later stages may contribute to an accelerated transition from Khalal to Rutab, potentially affecting fruit quality and texture.

### 3.4 Analysis of difference of environmental cues

To get a more concise overview of the environmental cues affecting date fruit ripening, we then focused on the differences between day and night average measurements for RH, temperature, and UV index across four date palm fruit stages (Hababouk, Kimri, Khalal, and Rutab) from 2019 to 2024. Instead of tracking each year individually, we focused on the overall trends and variations across the stages.

#### 3.4.1 Temperature

**Figure 6:**
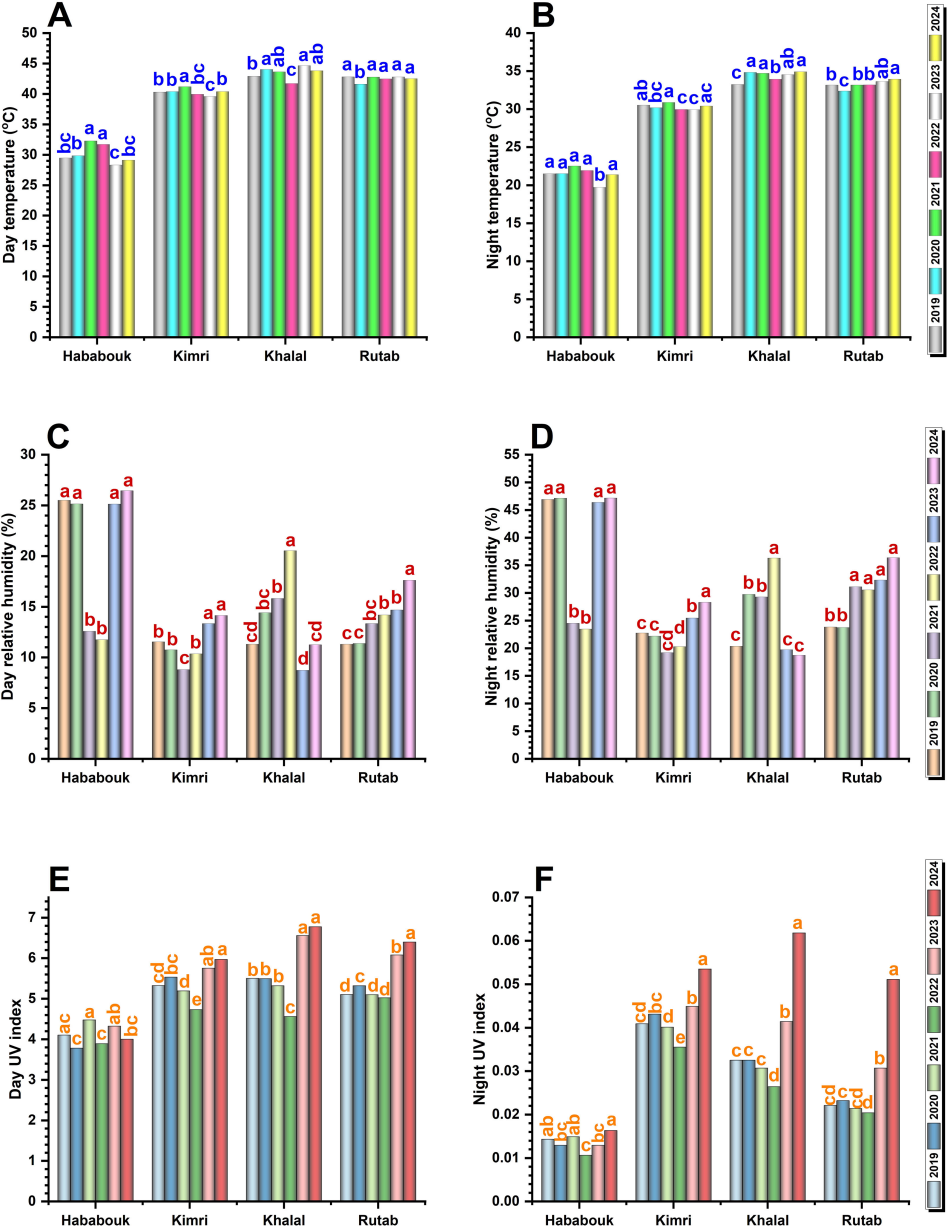
Comparison of environmental cues during date palm fruit ripening stages (2019-2024) in Al-Ahsa, Saudi Arabia. A comparative analysis of day and night measurements for **(A, B)** temperature, **(C, D)** relative humidity (RH), and **(E, F)** solar UV index across four ripening stages: Hababouk, Kimri, Khalal, and Rutab. Different letters (a, b, c, d) above bars indicate statistically significant differences between years within each ripening stage at p < 0.05.

Temperature fluctuations played a crucial role in the ripening process and showed an inverse trend to humidity. Generally, day- and night-time temperatures showed a clear and significant increase from the Hababouk to the Khalal stages, peaking in Khalal, and then slightly decreasing in the Rutab stage (**Figures 6A, B**). There were some variations in peak temperatures between years, but the overall trend remained consistent. During the early Hababouk stage, day temperatures ranged between 28-33 °C and showed small fluctuations across years, with 2021 recording the highest values. At Kimri stage, both day- and nighttime temperature increased significantly (*p* ≤ 0.05), often reaching 39-41 °C and 29-31 °C, respectively. In the Khalal stage, temperatures continued to rise, and it witnessed the highest temperature, particularly in 2021 and 2023, which was notably higher than in 2019 and 2020, coinciding with lower RH levels. At the Rutab stage, daytime temperatures stabilized at 41-43 °C. However, night temperatures remained significantly (*p* ≤ 0.05) different, with lower values recorded in 2019 and 2020, while 2024 remained significantly (*p* ≤ 0.05) higher, indicating consistent warming trends over the years.

#### 3.4.2 Relative humidity

RH showed a clear decreasing trend as the fruit progresses from the Hababouk to the Khalal stage, followed by a slight increase in the Rutab stage (**Figures 6C, D**). There are noticeable fluctuations, with some years having higher humidity during the earlier stages (Hababouk and Kimri) and lower humidity during the later stages (Khalal and Rutab). The range of variation appears to be wider in the earlier stages. Generally, nighttime RH was generally higher than daytime humidity and exhibited less dramatic fluctuations across the stages. During the Hababouk stage, both day and night RH are at their highest, ranging from 25-30% and 47-48%, respectively. Notably, during 2021 and 2022, RH levels dropped significantly (*p* ≤ 0.05), while for the remaining years, RH fluctuations were non-significant. However, as the fruit transitions into the Kimri stage, a notable and significant reduction in RH was observed, with day RH dropping as low as 8-15%, and night RH levels to 20-30%. The Khalal stage experienced the significantly (*p* ≤ 0.05) lowest day RH (9 and 11%) and night RH (19 and 18%) during 2023 and 2024, which may contribute to premature dehydration and hinder proper ripening. By the Rutab stage, RH increased slightly, especially this increase was significant during 2024, indicating better moisture retention.

#### 3.4.3 UV index

The UV index showed significant year-wise fluctuations, particularly during the later (Khalal and Rutab) stages of ripening, indicating that these stages experienced the highest exposure to UV radiation (**Figures 6E, F**). In the Hababouk stage, the day UV index ranged from 3.8-4.6, while night UV levels remain negligible. The Kimri, Khalal, and Rutab stages experienced significantly (*p* ≤ 0.05) higher UV exposure, particularly during 2023 and 2024. At Kimri stage, the peaks reaching 5.6-6.0. The Khalal stage experienced the highest day UV exposure during 2023 and 2024, significantly (*p* ≤ 0.05) higher than previous years. The Rutab stage showed consistently high UV exposure, like the Khalal stage, with day UV values remaining significantly (*p* ≤ 0.05) higher in 2024, reaching 6.0-6.3. Night UV, although minimal, followed the same trend, with significant differences in recent years, with the highest value in 2024, reflecting an increase in radiation exposure in recent years.

Overall, the combination of rising temperatures, declining RH, and increasing UV exposure, especially in 2021 and 2024, suggests that environmental stressors may be accelerating fruit dehydration, affecting ripening process. These variations could contribute to the observed non-ripening issue in Saudi Arabian date palms, particularly by disrupting the balance between moisture retention and fruit softening. Addressing these environmental challenges through optimized irrigation and shading techniques may help mitigate the impact on fruit development phase change and quality.

### 3.5 Analysis of heat units and irradiance at rutab stage

**Figure 7:**
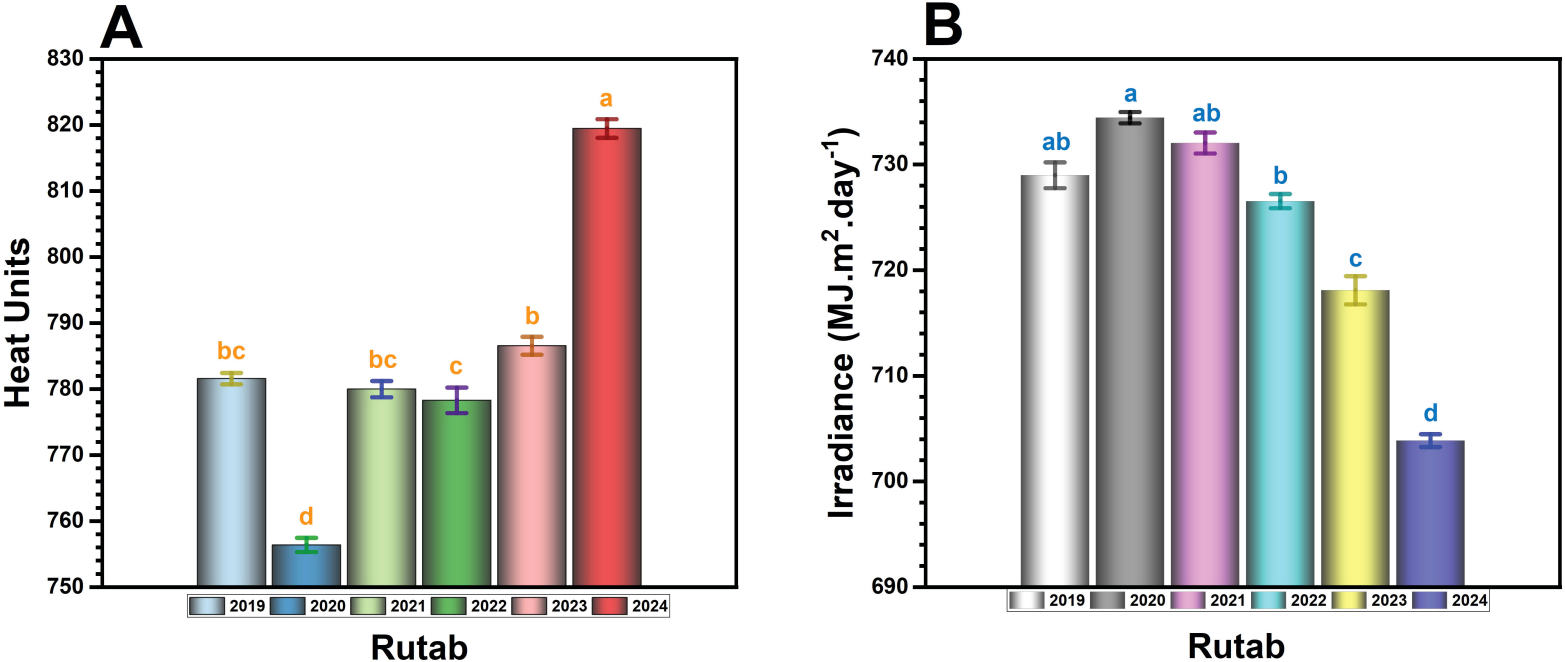
The annual variation in **(A)** total heat units and **(B)** total irradiance (MJ m^-2^ day^-^¹) during the Rutab ripening stage of date palm fruit in Al-Ahsa, Saudi Arabia, from 2019 to 2024. Bars represent yearly mean values. Different letters (a, b, c, d) above bars indicate statistically significant differences between years at Rutab stage at *p* < 0.05.

The total heat units (**Figure 7A**) showed a clear upward trend over the years, with the highest accumulation recorded in 2024 (~825 HU), which is significantly (*p* ≤ 0.05) higher than all previous years. The 2023 season (~795 HU) also exhibited an increase compared to earlier years, but the heat accumulation was still lower than in 2024. In contrast, 2020 had the lowest heat units (~755 HU), which was significantly (*p* ≤ 0.05) lower than all other years. Years like 2019, 2021, and 2022 (~775–785 HU) remained relatively stable without drastic fluctuations. The unprecedented heat accumulation in 2023 and 2024 may have contributed to incomplete ripening, as extreme temperatures can lead to hardened fruit texture, reduced sugar conversion, and premature drying, preventing fruit flesh softening.

Total irradiance during Rutab stage (**Figure 7B**) showed a progressive decline over the years, with the lowest values observed in 2024 (~700 MJ m^2^ day^-^¹), which is significantly (*p* ≤ 0.05) lower than all previous years. In contrast, 2019 and 2020 recorded the highest irradiance levels (~728–735 MJ m^2^ day^-^¹), supporting optimal ripening conditions. The decrease became more evident in 2023 (~710 MJ m^2^ day^-^¹), preceding the lowest values in 2024. Lower irradiance can reduce photosynthetic efficiency, leading to insufficient sugar accumulation and metabolic energy required for the final ripening stages. This decline, coupled with excessive heat units, creates an imbalance where fruits experience dehydration without the necessary energy to convert starch into sugars, leading to a failure to achieve the desired Rutab texture and sweetness.

### 3.6 Stage-specific effect sizes (η^2^) of environmental variables on date palm fruit development

**Table 1** presents the effect sizes (η^2^) obtained from ANOVA analyses to quantify the influence of various environmental variables on the developmental stages of date palm fruit, namely Hababouk, Kimri, Khalal, and Rutab. The η^2^ values indicate the proportion of variance in each developmental stage that are explained following by individual environmental factors. Notably, day and night UV index showed the largest effects during the Khalal and Rutab stages, with η^2^ values reaching as high as 0.85, suggesting a strong influence of UV exposure during the latter stages of fruit maturation. Heat units and irradiance also demonstrated substantial explanatory power during the Rutab stage (η^2^ = 0.97 and 0.91, respectively), indicating their critical role in the final ripening phase. In contrast, early stages such as Hababouk and Kimri generally showed lower η^2^ values across most variables, with the exception of day relative humidity (η^2^ = 0.32) and night relative humidity (η^2^ = 0.38) during Hababouk.

**Table 1:** Effect sizes (η^2^) from ANOVA for environmental variables across date palm fruit development stages Analysis of Variance (ANOVA).

Environmental variables	Hababouk	Kimri	Khalal	Rutab
Day temperature	η^2^ = 0.13	η^2^ = 0.02	η^2^ = 0.19	η^2^ = 0.09
Night temperature	η^2^ = 0.06	η^2^ = 0.01	η^2^ = 0.19	η^2^ = 0.11
Day relative humidity	η^2^ = 0.32	η^2^ = 0.12	η^2^ = 0.21	η^2^ = 0.18
Night relative humidity	η^2^ = 0.38	η^2^ = 0.09	η^2^ = 0.24	η^2^ = 0.14
Day UV index	η^2^ = 0.07	η^2^ = 0.19	η^2^ = 0.72	η^2^ = 0.65
Night UV index	η^2^ = 0.06	η^2^ = 0.22	η^2^ = 0.85	η^2^ = 0.85
Heat units	–	–	–	η^2^ = 0.97
Irradiance	–	–	–	η^2^ = 0.91

Day and night temperatures exhibited relatively modest effect sizes (η^2^) across the date palm fruit developmental stages. The influence of day temperature was more pronounced during the Khalal stage (η^2^ = 0.19), suggesting a moderate role in fruit maturation, while its effect was minimal during the Kimri stage (η^2^ = 0.02). Similarly, night temperature showed limited influence overall, with slightly higher η^2^ values observed during Khalal (η^2^ = 0.19) and Rutab (η^2^ = 0.11) stages. These results indicate that temperature, particularly during the day, may contribute to fruit development, but its effect is less substantial compared to other environmental variables such as UV index or humidity. These findings suggest that environmental factors, particularly UV radiation, heat accumulation, and humidity, have stage-specific effects on date palm fruit development, with their impact intensifying during the later stages of ripening.

## 4 Discussion

The date palm, a cornerstone of life in the Arabian Peninsula, has provided sustenance for millennia. However, recent climate changes, particularly in Saudi Arabia, pose a significant threat to date palm cultivation, due to a recently emerged DFUS. The environmental stressors, exhibiting fruit development stage-specific variations, especially during the crucial Khalal and Rutab stages, can disrupt fruit metabolism and lead to irregular ripening. The situation is likely to worsen, as climate projections indicate a global temperature rise of approximately 1.5 °C by 2030, 2.0 °C by 2050, and 3.0 °C by 2070 (IPCC, 2023). Arid regions like Saudi Arabia are predicted to experience even more pronounced warming, potentially exceeding 2.0 °C by 2030, 2.5-3.5 °C by 2050, and 3.5-4.5 °C by 2070 (Almazroui, 2013). Such pronounced warming likely disrupts ethylene/ABA pathways, contributing to DFUS. Projected increases of 1.5 °C could elevate peak temperatures to 49.5–50 °C, while a 4.5 °C rise may push them to 52.5–53 °C, amplifying heat stress and ripening inhibition, consistent with regional climate trends (Sharif, 2015). The present study aimed to identify the specific environmental triggers of DFUS and investigate how their complex interactions disrupt the physiological, biochemical, and molecular mechanisms governing fruit maturation, ultimately impacting yield and quality of this vital crop.

Overall, DFUS significantly (*p* ≤ 0.05) impacts prominent date palm cultivars, including Khalas (40%), Reziz (19%), Sheshi (16%), Ghur (16%), Shahal (10%), and several others, with its prevalence varying across different regions. Interestingly, two of our previous studies focusing on Khalas, Reziz, and Sheshi revealed that Khalas employed the highest number of drought-responsive genetic resources (ESTs) to withstand drought conditions, both under controlled and natural environments (Alhajhoj et al., 2022; Iqbal and Munir, 2024). Despite this robust drought adaptation, Khalas has been the most severely affected by DFUS. This paradox raises important questions about the underlying causes, warranting a comprehensive study to uncover the precise mechanisms driving this phenomenon. One possible explanation is that Khalas, being native to desert climate regions such as Al-Ahsa and Al-Qassim, has evolved and acquired genetic adaptations specifically tailored to cope with drought stress. However, the recent and rapid changes in climate conditions—characterized by extreme temperature fluctuations, increased UV exposure, and erratic humidity levels—may have exposed vulnerabilities in Khalas that were previously unaccounted for. The paradoxical vulnerability of the drought-adapted Khalas cultivar to DFUS, despite its resilience, may stem from specific gene-environment mismatches under combined heat and UV stress. Hypothesizing, elevated temperatures (e.g., 48 °C peaks) and UV exposure could downregulate ethylene biosynthesis genes (e.g., ACS, ACO), critical for ripening, while overactivating ABA-related genes (e.g., NCED1) under drought-like conditions, disrupting the ethylene/ABA balance. This mismatch, unique to Khalas under intensified stressors, may impair fruit softening and sugar accumulation, warranting further molecular analysis to confirm. Our investigation into the interplay of temperature, RH, heat units, UV index, and irradiance revealed a compelling narrative of environmental influence on fruit ripening. Temperature, a master regulator of fruit development (Adams et al., 2001), fluctuated dramatically across the six years studied, with peak daytime temperatures soaring to 48 °C during the critical Kimri and Khalal stages (**Figure 5**). These thermal extremes pose a significant threat of heat stress, capable of disrupting the delicate enzymatic machinery and metabolic pathways vital for natural fruit ripening (Haider et al., 2013; Li et al., 2021). While daytime temperatures ranged from 24 °C to 38 °C during the Hababouk stage and night temperatures varied between 14 °C and 28 °C, these fluctuations already hint at the challenges faced by the developing fruit after fruit setting. The dynamic thermal environment is further underscored by the temperature ranges observed across subsequent stages: 24-38 °C (Hababouk), escalating to 48 °C (Kimri), fluctuating between 36-48 °C (Khalal), and finally settling between 38-46 °C (Rutab). Similarly, night-time temperatures during Hababouk (14-28 °C), Kimri (18-34 °C), Khalal (29-38 °C), and Rutab (30-37 °C), contribute to cumulative thermal stress on fruit ripening process. Intriguingly, 2024 presented a different picture: while temperatures remained high, they exhibited linear stability, particularly during the crucial Khalal and Rutab phases. This stability correlates with conditions conducive to enhanced sugar accumulation and fruit softening (Alam et al., 2023), suggesting a potential positive shift in climatic patterns. Temperature alone may not be the primary driver of DFUS; rather, its impact is likely modulated by interactions with other environmental factors such as humidity, solar radiation, and soil conditions. Studies have shown that multi-factorial stress—rather than isolated temperature fluctuations—can significantly (*p* ≤ 0.05) disrupt fruit ripening physiology (Espley and Jaakola, 2023; Sharma et al., 2023; Zhu et al., 2023). For instance, elevated night-time humidity or low vapor pressure deficit can hinder sugar accumulation and color development (Durán-Soria et al., 2020). These combined stresses may account for the unexpectedly high DFUS incidence in 2024, despite overall stable temperature conditions during 2024. However, our analysis extends beyond mere temperature fluctuations, and we tracked total heat unit accumulation and total irradiance levels during the Rutab stage. A gradual increase in heat units from 2019 to 2024, coupled with a simultaneous decline in total irradiance, demonstrated a complex picture that might imbalanced photosynthesis system/photosynthetic capacity and fruit ripening processes. Similar results have been achieved in jujube, when increased temperature led to fruit ripening with higher anthocyanin contents but reduction in soil moisture (30-50% of field capacity) at the same higher temperature reduced the anthocyanin but increased chlorophyll levels (Jiang et al., 2020). The convergence of these factors – fluctuating temperatures, increasing heat units, and decreasing irradiance – likely exerts a powerful and multifaceted influence on enzymatic activities, metabolic processes, and ultimately, the successful progression of date palm fruit ripening. Such extreme temperatures can induce heat stress, disrupting enzymatic activity, altering sugar metabolism, and negatively affecting fruit quality (Hasanuzzaman et al., 2013). Indeed, prior research has shown that excessive heat can lead to irregular ripening (Antunes and Sfakiotakis, 2000; Sharma et al., 2023), and our findings support this, as the years with the highest temperature fluctuations also showed the greatest potential for heat stress during the critical Khalal and Rutab stages, which are crucial for fruit maturation and sugar accumulation (Alam et al., 2023).

The inter-annual variability in RH significantly (*p* ≤ 0.05) impacted date palm fruit growth and ripening. Our findings revealed that the Khalal stage, marked by extreme RH fluctuations, a period of rapid growth and color change, is exquisitely sensitive to RH fluctuations. Low RH during this stage can impair water retention and physiological processes, leading to suboptimal fruit development (Jain et al., 2023). In contrast, linear and reduced RH levels observed in 2019–2022 likely enhanced fruit quality during the Rutab stage by supporting uniform sugar accumulation and texture development (Ismail et al., 2023). Nonetheless, higher RH levels with certain peaks during the Rutab stage in 2023 and 2024 may have a negative impact on fruit ripening. High humidity can disrupt date palm fruit ripening, especially during the Khalal and Rutab stages (**Figures 6A, B**), by hindering the necessary moisture loss for optimal texture, sugar concentration, and quality (Siddiqui, 2015; Khodabakhshian and Khojastehpour, 2021). This can also interfere with sugar accumulation affecting sweetness (Alam et al., 2023), impair color and texture development, and promote fungal and bacterial growth, leading to spoilage (Siddiq and Greiby, 2013). High humidity also exacerbates the negative impacts of other stressors like high temperatures, increasing the risk of physiological disorders and reducing overall fruit quality.

The intensifying solar UV radiation in Al-Ahsa, particularly during the critical Kimri, Khalal, and Rutab stages of date palm fruit development, may have impacted the ripening process. The analysis of solar UV index and high η^2^ values (up to 0.85) suggested that elevated UV levels, especially during 2023 and 2024, may have exacerbated the non-ripening in Khalas cultivar in Al-Ahsa and possibly other regions in Saudi Arabia. UV exposure, especially UV-B and UV-C, can induce oxidative stress within fruit tissues by generating reactive oxygen species (ROS), such as superoxide radicals and hydrogen peroxide, which damage cellular components like lipids, proteins, and DNA (Rastogi et al., 2010). This oxidative damage is evidenced by increased levels of lipid peroxidation products like malondialdehyde (MDA) and altered activities of antioxidant enzymes (González-Aguilar et al., 2001). Beyond direct cellular damage, high UV levels can interfere with critical hormonal pathways that regulate ripening. While some studies suggest low doses of UV-C can stimulate ethylene production postharvest in fruits like tomato and strawberry, potentially influencing ripening processes (Tiecher et al., 2013), prolonged or intense pre-harvest UV exposure may lead to detrimental dysregulation. Perhaps most concerning, high UV exposure has been shown to disrupt the intricate hormonal signaling pathways involving ethylene and ABA, which orchestrate the complex choreography of ripening (Serrano et al., 2001; Yahia and Kader, 2011; Severo et al., 2015; Shareef and Al-Khayri, 2020). Additionally, UV irradiation (UV-C) could delay ripening by slowing starch conversion and maintaining firmness (Naradisorn, 2021); while also enhancing total phenolic content, influencing carotenoid accumulation, and antioxidant activity which could hinder ripening progresses (Ding et al., 2015). Such hormonal imbalances can lead to uneven or delayed ripening, compromising fruit quality and potentially increasing susceptibility to postharvest decay. The combined effect of these UV-induced disruptions painted a picture of a ripening process under duress, potentially impacting the overall quality, texture, and longevity of the date crop in this region.

Overall date palm fruit ripening is a complex, orchestrated process involving intricate interactions between transcription factors (TFs), hormones, and signaling pathways (**Figure 1**). Key TFs like NAC, NOR, and ERFs regulate gene expressions driving ripening-related changes. Hormones, including ethylene, auxins, gibberellins (GA), ABA, and jasmonic acid (JA), play crucial roles in this regulation (Giovannoni, 2001; Serrano et al., 2001; Awad et al., 2011; Yahia and Kader, 2011). Ethylene, synthesized from methionine via the Yang cycle and ACC synthase (ACS), is particularly prominent in promoting ripening. ABA, potentially influencing stress responses, and JA, possibly modulating defense mechanisms, also contribute. Cell wall degradation, facilitated by enzymes like polygalacturonase (PG) and cellulase, leads to fruit softening (Elbar et al., 2023; Tipu and Sherif, 2024). Pigment accumulation, including carotenoids and anthocyanins, imparts characteristic color changes, while sugar accumulation contributes to sweetness (Carbone et al., 2009; Shareef et al., 2020). Environmental cues, such as temperature, relative humidity, UV light, and irradiance, perceived as exogenous signals, further modulate the ripening process (**Figure 1**). This network of positive and negative regulatory mechanisms ensures the coordinated progression of physiological and biochemical changes leading to a ripe, palatable fruit.

**Figure 8:**
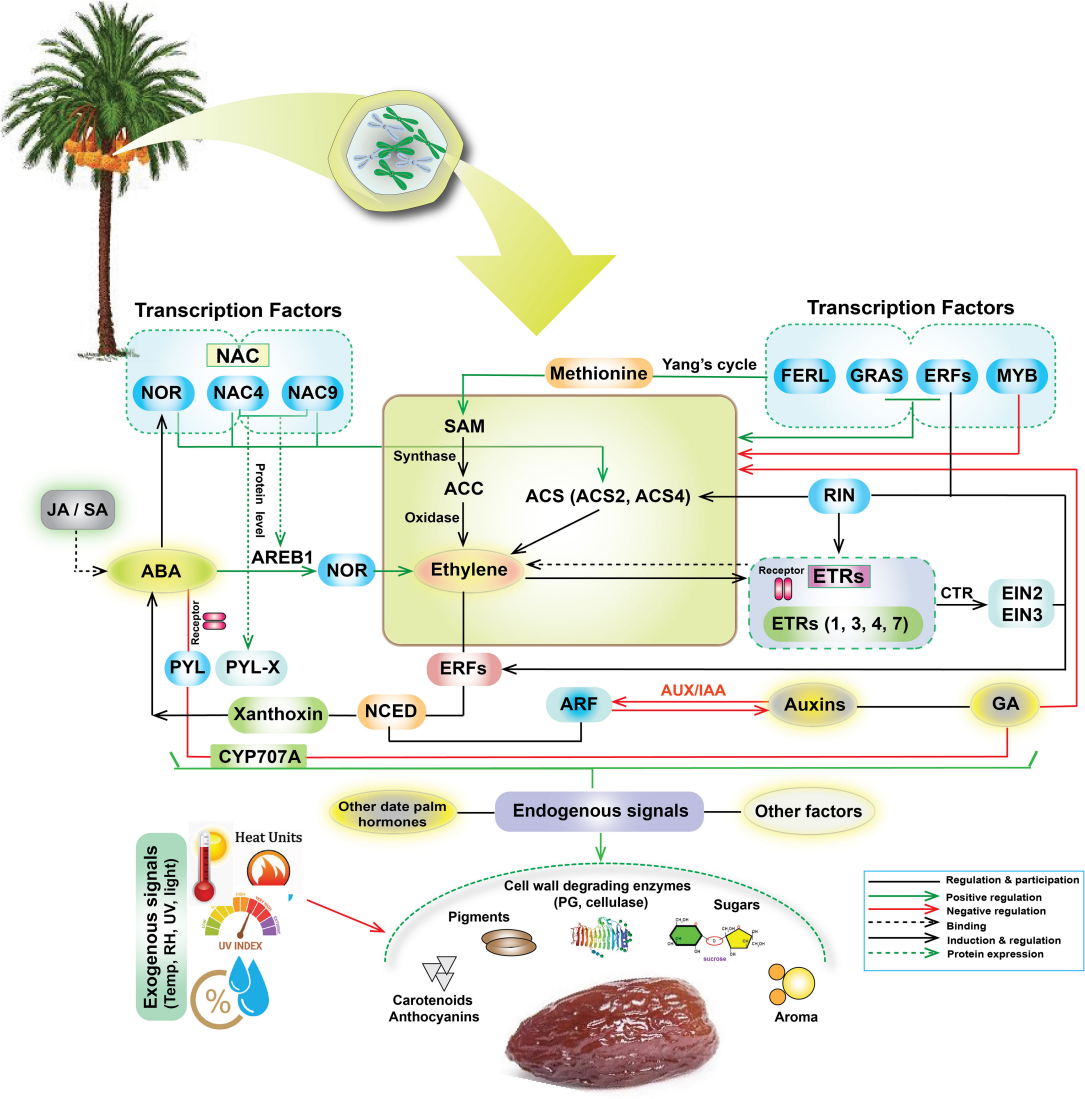
Potential integrated molecular mechanisms depicting the complex interplay of transcription factors (NAC, NOR, ERFs, GRAS, MYB, RIN), hormones (ABA, ethylene, auxins, GA, JA/SA), and signaling pathways and environmental factors governing date fruit ripening. Central processes include the Yang cycle, ACC synthase activity, and ethylene production, along with ABA’s influence via AREB1 and feedback loops involving CYP707A and NCED. Downstream targets include cell wall degradation (PG, cellulase), pigment accumulation (carotenoids, anthocyanins), sugar production, and aroma development. These pathways are modulated by both endogenous (other date palm hormones) and exogenous (temperature, relative humidity (RH), UV light, light intensity) signals.

The biochemical attributes of date palm fruit ripening, as illustrated in **Figures 1** and **8**, are intricately regulated by a complex interplay of hormones, TFs, and metabolic pathways. However, environmental stressors such as high and fluctuating temperatures, elevated UV index, and erratic RH can disrupt these processes, leading to the DFUS, where fruits fail to ripen properly. During the Khalal stage, key biochemical changes such as sugar accumulation, anthocyanin synthesis, and hormone regulation (e.g., ABA, ethylene, and auxins) are critical for transitioning to the Rutab and Tamar stages (**Figure 8**).

High temperatures, particularly during the Khalal and Rutab stage, can impair the activity of enzymes like CYP707A and NCED (Toh et al., 2008; Zandalinas et al., 2016), which are involved in ABA metabolism, thereby delaying ripening (Carbone et al., 2009). Additionally, excessive heat units and UV exposure can disrupt ethylene biosynthesis pathways, including the conversion of SAM to ACC by ACC synthase, leading to irregular ethylene production and impaired fruit softening (Alam et al., 2023). Fluctuating RH levels further exacerbate these issues by altering water retention and metabolic activities, particularly during the Khalal and Rutab stages, where low RH (or high temperature) can reduce sugar accumulation and anthocyanin synthesis, contributing to the DFUS problem (Tan et al., 2023). Reduced irradiance, often coupled with high UV exposure, can also impact transcription factors like NAC, NOR, and MYB, which regulate ripening-related genes. For instance, high UV levels may down-regulate NAC4 and NAC3, delaying the transition from Khalal to Rutab (Kalantari, 2004). Furthermore, the interplay of these environmental stressors can disrupt auxin signaling pathways, mediated by ARF and AUX/IAA, leading to imbalances in fruit growth and ripening. Additionally, interactions among these environmental factors influence the expression of MADS-box transcription factors like RIN (ripening inhibitor), which coordinates ripening pathways by activating genes involved in ethylene biosynthesis, cell wall modification, and sugar metabolism. RIN integrates external cues to fine-tune ripening (Li et al., 2020). Furthermore, AP2/ERF family members regulate downstream responses to environmental signals, modulating gene networks based on external conditions (Gao et al., 2020).

So, environmental stress likely triggers DFUS by disrupting hormone-regulated ripening pathways, especially those involving ABA and ethylene. Normally, increased ABA and ethylene biosynthesis activates genes controlling sugar accumulation, pigmentation, and softening. Stress conditions such as salinity, drought, or high humidity downregulate key biosynthetic genes like NCED (ABA) and ACS/ACO (ethylene), delaying ripening (Rosca et al., 2023; Bianchetti et al., 2024). This imbalance also suppresses ripening-related transcription factors such as EIL (Ethylene Insensitive-Like) and ERF (Ethylene Response Factors), further impairing development (Yin et al., 2010). The result is a physiological block in ripening—manifested as color retention, low sugar, and firm texture—despite normal fruit growth. It is noteworthy to mention that while this study establishes stage-specific environmental impacts (e.g., UV η^2^ = 0.85–0.97 during Khalal/Rutab), direct correlations with yield/DFUS incidence could not be assessed due to unavailable multi-year field records (2019–2024) and our prioritized focus on identifying environmental DFUS triggers. Nevertheless, the high effect sizes for UV, heat units (>800 HU), and low humidity (<20% RH) during critical stages—aligned mechanistically with known stress physiology (UV-impaired ethylene signaling; heat-reduced sugar conversion; humidity-driven dehydration)—strongly support their biological relevance in ripening failure. Future work must integrate longitudinal yield/DFUS data with environmental monitoring to validate these relationships.

## 5 Conclusions

The present study demonstrated that temperature, relative humidity, UV radiation, heat unit accumulation, and irradiance collectively shaped the ripening trajectory of date palm fruit in Al-Ahsa, with their effects varying by developmental stage. The Khalal and Rutab stages emerged as the most sensitive to environmental stressors, where elevated daytime temperatures (often >48 °C), sharp declines in RH (<15% day/<20% night), and intensified UV exposure (particularly in 2023–2024) significantly coincided with delayed or incomplete ripening. Increasing cumulative heat units in recent years, coupled with declining irradiance, suggested a growing imbalance between metabolic energy requirements and photosynthetic capacity, which predisposed fruits to dehydration without adequate sugar conversion. While temperature alone was not the dominant driver, its interaction with low RH and high UV—especially under combined heat–UV stress—appeared to disrupt ethylene/ABA homeostasis, impairing softening and sweetness. The observed trends indicated that climate-driven environmental extremes were intensifying and could have exacerbated the “dark-flesh, unripe-surface” (DFUS) disorder in susceptible cultivars like Khalas. These findings underscored the need for adaptive orchard management strategies, such as microclimate modification, optimized irrigation scheduling, and protective shading, to mitigate environmental stress and safeguard date palm fruit quality in a warming, increasingly UV-intense environment.

## Data Availability

The original contributions presented in the study are included in the article/supplementary material. Further inquiries can be directed to the corresponding author.
